# Tattoo for Sheep Marking

**Published:** 1891-12

**Authors:** J. B. Hellier


### TATTOO FOR SHEEP MARKING.

By J. B. HELLIER.

This way of marking stud sheep for the purpose of keeping up the register of their pedigrees, has been in use in the stud flocks of Europe, America, and Australia for many years past. The tattoo method is now, however, coming into use, for marking sheep as a guide to ownership and identification. The ordinary way of tattooing stud sheep is to put the tattoo mark or number on the ear. The instrument used for this purpose is a small tongs-like implement, the figures or letters being fixed in one blade or side of the tongs. The letters are pricked into the ear, and soot or India ink rubbed in, so that there should be formed a black tattooed letter on the ear.

It is now proposed to mark the sheep under similar regulations as those of the Registered Brands Act, the tattoo marks to be put on in the following order, to indicate ownership :

| | |
|---|---|
| First mark, - - - | The near or left ear. |
| Second mark, - - - | The off or right ear. |
| Third mark, - - - | Under side of the tail. |
| Fourth mark, - - - | Under the near fore arm. |

The last tattoo mark to be considered the mark of the present owner, as in the case of the succession of brands under the Registration of Brands Act.

It has been found that if the letters are stamped on an ink pad, just like the stamps are inked at the post office that the tattooing is effective, and the letters and figures become as indelible as when the ink or soot is rubbed in, in the usual way.

The tattoo stamp can be used without the tongs or pliers, and a private mark made on any sheep which would secure the identification of the sheep or their skins even when the ears are cut off, the sheep shorn, or the brand mark blotched or defaced.

The great similarity of a great number of ear marks, too, and the ease with which they can be altered, or, as the Australians say, *faked*, often stands in the way of easy and certain identification.

For the protection of the tattoo mark on the ear of the sheep, any wilful disfigurement of the mark involves a penalty not exceeding £100, and a thief who cuts off the sheep's ear is severely punished. In fact, where the tattoo is used as a brand mark, sheep or sheep skins without ears are deemed subjects for strict police investigation.

Anyone who has to identify sheep and goats and their skins will see at once the value of tattoo marking ; and I think its general adoption would aid in the detection of thefts, and everything which facilitates the catching and conviction of thieves is an additional help to the putting down of stock stealing.—(*Agricultural Journal,* Cape Colony.)

